# The quality and antioxidant elucidation of germinated flaxseed treated with acidic electrolyzed water

**DOI:** 10.1002/fsn3.2538

**Published:** 2021-09-10

**Authors:** Shasha Huang, Haicheng Zhang, Xiaopeng Qin, Chengzhen Nie, Xiao Yu, Qianchun Deng

**Affiliations:** ^1^ College of Food and Bioengineering Zhengzhou University of Light Industry Henan Key Laboratory of Cold Chain Food Quality and Safety Control, and Collaborative Innovation Center for Food Production and Safety Zhengzhou China; ^2^ Oil Crops Research Institute Chinese Academy of Agricultural Sciences Hubei Key Laboratory of Lipid Chemistry and Nutrition, and Key Laboratory of Oilseeds Processing, Ministry of Agriculture Wuhan China

**Keywords:** acidic electrolyzed water, antioxidant properties, flaxseed, germination, lipid concomitants

## Abstract

The endogenous fortification of antioxidant lipid concomitants in flaxseed was imperative to improve the oxidative stability of α‐linolenic acid (ALA) in flaxseed and flaxseed oil upon processing, storage and gastrointestinal digestion. The comparative effects of acidic electrolyzed water (ACEW) and tap water (TW) on the triglyceride configuration, typical lipid concomitants, and antioxidant properties of flaxseed were conducted during 0–5 days of germination. The results showed that ACEW enhanced the germination rate of flaxseed by 18.25% and simultaneously suppressed the dynamic depletion of ALA by 5.32% when compared with TW (*p* < .05). The total phenolic acids, lignans, and flavonoids were effectively accumulated in flaxseed following ACEW‐mediated germination with the further increase by 4.82%, 15.48%, and 8.22% in comparison with those induced by TW (*p* < .05). The total contents of cyclolinopeptides in flaxseed progressively dropped following either ACEW or TW treatment, a slighter decrease by 5.59% for flaxseed treated by ACEW than that by TW. Notably, the maximum accumulation of tocopherols and phytosterols had been early obtained for flaxseed treated with ACEW for 2–3 days due to the de novo synthesis or intermolecular conformational transition (*p* < .05). Most importantly, ACEW‐mediated germination led to higher increment of the thermal oxidative stability and antioxidant properties of flaxseed and flaxseed oil in comparison to TW. In brief, the initial oxidation temperature increased by 7.09% and 3.06% (*p* < .05), and the antioxidant activities as evaluated by DPPH, ABTS, and FRAP values raised by 3.86%–28.07% and 4.21%–9.18% (*p* < .05), respectively. These findings clarify that the germination especailly mediated by ACEW could be an effective method to further optimize the nutritional and functional properties of flaxseed through reconstructing the endogenous antioxidant system.

## Abstract 2

The endogenous fortification of bioactive lipid concomitants in flaxseed was imperative to improve the oxidative stability of α‐linolenic acid in flaxseed and flaxseed oil upon processing, storage, and gastrointestinal digestion. The comparative effects of acidic electrolyzed water (ACEW) and tap water (TW) on the changes in triglyceride configuration, typical lipid concomitants, and antioxidant properties of flaxseed and flaxseed oil were conducted during 5 days of germination. Our findings clarify that the germination especailly mediated by ACEW could be an effective method to further optimize the nutritional and functional properties of flaxseed through reconstructing the endogenous antioxidant system.

## 1 INTRODUCTION

Flaxseed was rich in planted‐derived n‐3 polyunsaturated fatty acid (n‐3PUFA) α‐linolenic acid (ALA, ~59%), which possessed cardiovascular‐protective, anti‐cancer, anti‐inflammatory, neuro‐protective properties, etc. (Parikh et al., 2019; Yu et al., 2018). However, the oxidation of ALA was easily initiated upon the gastrointestinal digestion, which intimately depended on the oxidative degree of flaxseed oil during the processing and storage (Nieva‐Echevarria et al., 2017). Fortunately, the simultaneous intake of natural antioxidants, such as phenolic compounds, effectively suppressed the PUFA oxidation (Pilar et al., 2017). These indicated that the endogenous fortification of antioxidant lipid concomitants in flaxseed, especially their migration into oil phase, was profitable and imperative to restrain the oxidative susceptibility of ALA upon processing, storage, and gastrointestinal conditions.

Indeed, flaxseed contained bioactive lignans, phenolic acids, flavonoids, tocopherols, phytosterols, cyclolinopeptides (CLs), etc. (Shim et al., 2014). The phenolic compounds and tocopherols were considered as the main components that reflected the antioxidant capacities of flaxseed and flaxseed oil (Deng et al., 2018). Regrettably, the naturally occurring tocopherols in flaxseed mainly existed in form of γ‐tocopherol (>90%) but not α‐tocopherol, which partly limited its antioxidant potential (Li et al., 2019). Meanwhile, the phenolic acids and flavonoids in native flaxseed were only marginally migrated into the oil phase following cold‐pressed extraction, which was still limitedly improved upon the thermal pretreatment of flaxseed due to the relative deficiency and natural hydrophilicity (Suri et al., 2020). The lignan secoisolariciresinol diglucoside (SDG) was considered as a key component to protect ALA‐rich oil bodies in flaxseed against environmental stress during seed development and dormancy owing to its oligomeric structure (Nikiforidis, 2019; Toure & Xu, 2010). Although the contents of phytosterols in flaxseed were relatively high, the antioxidant activity of primary *β*‐sitosterol was apparently weaker than that of minor stigmasterol (Singh, 2013). Flaxseed contained abundant CLs, which possessed relatively weak antioxidant activities, but also contributed to the bitter off‐taste of flaxseed oil (Bruhl et al., 2007; Zou et al., 2018). Therefore, a suitable pretreatment method should be explored in order to further fortify the accumulation of antioxidant lipid concomitants in flaxseed based on the de novo synthesis or intermolecular conformational transition without obviously changing the contents of bioactive ALA in flaxseed.

Previous studies had illustrated that the accumulation of lignans, phenolic acids, and flavonoids, as well as the antioxidant activities, subjected to varying degrees of promotion following flaxseed germination treated with tap water (TW) (Li et al., 2019; Wang et al., 2015). It also had been found that the contents of tocopherols in flaxseed significantly increased after 1–2 days of germination (Li et al., 2019). Although direct evidence has yet to be sought, Shi et al., (2010) had reported that the levels of total phytosterols in soybeans increased by onefold during 3 days of germination. Regrettably, no definite data had been available on the developmental changes of the CLs, as well as the ALA‐rich triglyceride (TAG) during flaxseed germination. The electrolyzed water is produced from the electrolysis of electrolyte solution and divided into acidic and alkaline electrolytic water according to the differences in pH value and available chlorine concentration (ACC) (Liu et al., 2013). Usually, the low‐concentration acidic electrolyzed water (ACEW) had been used for fresh‐cut lettuce and fresh organic broccoli due to its sanitization effect (Liu et al., 2017; Zhang & Yang, 2017). Notably, the previous study had found that ACEW was more effective than TW on improving the germination of brown rice and buckwheat, which might partly be attributed to its antibacterial activity (Hao et al., 2016; Liu et al., 2013). However, it was still unknown whether the promotive germination induced by ACEW was related to the enhanced accumulation of bioactive phytochemicals in flaxseed. Based on above, the comparative analysis of TAG configuration, bioactive lipid concomitants, and antioxidant properties of flaxseed sprouts initiated by ACEW and TW had been conducted, in order to obtain the flaxseed and flaxseed oil with optimal nutritional and functional properties.

## 2 MATERIALS AND METHODS

### 2.1 Preparation of acidic electrolyzed water

ACEW was prepared using XYS‐C‐12 Electric Sterilizing Water Generator (Baoji xinyuguang electromechanical Co., Ltd., Baoji, China). Briefly, the sodium chloride solution (0.5‰, m/v) was electrolyzed for 8 min under the operating current of 6.1 A and voltage of 8 V, respectively. The pH value of ACEW was measured using a pH meter (Model PHS‐3E, Shanghai, China). The ACC concentration was measured by iodometry method. The pH and ACC values of ACEW in this study were 3.50 ± 0.05 and 35.30 ± 3.25 mg/L, respectively.

### 2.2 Germination of flaxseed

The germination of flaxseed (Jinya 7#) was performed according to the method reported by Wang et al., (2015) with slight modification. Briefly, flaxseed was soaked in TW and ACEW for 30 min, evenly spread into sprouter (150 × 180 mm; 115 ± 5 g/plate, wet weight), and then placed in a constant temperature and humidity incubator (25 ± 2℃; 75 ± 5%) for 0–5 days, respectively. Flaxseed spouts were sprayed every 8–10 hr with the corresponding TW and ACEW, collected every day, freeze‐dried, and stored at –20℃ for further analysis.

### 2.3 Morphology observation of flaxseed

The morphological changes of flaxseed spouts were observed during 5 days of germination, and the pictures were taken using a Canon EOS 90D digital camera.

### 2.4 Analysis of polar lipid concomitants in flaxseed

#### 2.4.1 Extraction of phenolic compounds

Briefly, 0.3 g of freeze‐dried and crushed flaxseed sprouts was extracted with 5 ml of ethanol–water (80:20, v/v) using vortex mixing for 10 min coupled with ultrasonic bath for 15 min at 25℃ and then centrifuged at 6800 g for 10 min. The resulting supernatant was combined and stored at 4℃ in the dark until used.

#### 2.4.2 Total phenolic acids and flavonoids

The contents of total phenolic acids and flavonoids in flaxseed sprouts were determined by using the Folin–Ciocalteu colorimetric method and aluminum nitrate method, respectively (Yu et al., 2020). The results were expressed as milligram gallic acid equivalents (GAE) and rutin/100 g of flaxseed (dry basis), respectively. The free phenolic acids were further analyzed using an Agilent 1290 ultraperformance liquid chromatograph (UPLC) (Agilent, California, USA) equipped with a photodiode array(PDA) detector (Cong et al., 2020). Chromatographic separation was performed on an ACQUITY UPLC® BEH Shield RP18 column (2.1 × 100 mm, 1.7 μm). Elution was conducted with a two‐buffer gradient system at a flow rate of 0.21 ml/min: solvent A, 0.2% (v/v) acetic acid in water; solvent B, 100% methanol; a linear gradient (95%–75% A, 0–7.4 min; 75%–71% A, 7.4–10.07 min; 71%–64% A, 10.07–16.73 min; 64%–55% A, 16.73–23.4 min; 55%–35% A, 23.4–25.4 min; 35%–95% A, 25.4–27.4 min; 95% A, 27.4–35.0 min). The detector wavelength was set at 280 nm, and the injection volume was 3 μl. The individual free phenolic acids were quantified using the external standard method (for gallic acid, *y* = 33.302x‐5.4628; for ferulic acid, *y* = 4.8734x‐4.055; for syringic acid, *y* = 42.212x‐12.634; for *p*‐coumaric acid, *y* = 65.549x‐35.621; for caffeic acid, *y* = 40.673x‐8.524; for protocatechuic acid, *y* = 22.489x‐7.903; for vanillin, *y* = 54.607x‐18.281; for 4‐hydroxybenzoic acid, *y* = 8.5213x‐12.384).

#### 2.4.3 Lignans

The contents of lignan alkaline hydrolysates in flaxseed sprouts, including SDG, *p*‐coumaric glucoside (*p*‐CouAG), and ferulic acid glucoside (FeAG), were measured as previously described with slight modification (Deng et al., 2018). Briefly, the extracts were subjected to alkaline hydrolysis with the final NaOH concentration of 20 mM for 12 hr at 50℃ and then centrifuged at 4863 g for 10 min. The supernatant of hydrolytic reaction mixture was neutralized with dilute HCl solution and directly sampled to Agilent 1,290 UPLC system equipped with DAD detector and BEH C18 column (1.7 μm, 100 mm × 2.1 mm) maintained at 35℃. Elution was conducted with a two‐buffer gradient system at a flow rate of 0.20 ml/min (solvent A, 100% methanol; solvent B, 0.5% acetic acid aqueous solution): 0–8 min, 15% A; 8–16 min, 15%–28% A; 16–24 min, 28%–55% A; 24–32 min, 55%–85% A; 32–33 min, 85%–15% A; 33–35 min, 15% A. The detector wavelength was set at 200–400 nm, and the injection volume was 2.0 μl. The SDG and *p*‐CouAG were quantified using an external standard method (for SDG, *y* = 1433x+249.1; for *p*‐CouAG, *y* = 15086x+16258). The FeAG content was expressed as the ferulic acid equivalent following the molecular weight conversion between ferulic acid and FeA.

#### 2.4.4 Cyclolinopeptides

The contents of CLs in flaxseed sprouts were conducted as described previously with a slight modification (Lao et al., 2014). Briefly, the extracts were subjected to HPLC system with a Phenomenex Kinetex Pheny‐Hexyl analytical column (2.6 μm, 150 mm×4.6 mm) maintained at 35℃. Elution was conducted with a two‐buffer gradient system at a flow rate of 1.0 ml/min (solvent A, deionized water; solvent B, acetonitrile): 40%–90% B at 0–25 min, 90% B at 25–40 min, 100% B at 40–60 min. The detector wavelength was set at 214 nm, and the injection volume was 5.0 μl. The CLs were identified by comparing with the chromatograms as previously reported by Lao et al., (2014) and quantified relative to the external standard CL‐E.

### 2.5 Analysis of nonpolar tocopherols and phytosterols in flaxseed

According to Deng et al., (2018), the contents of tocopherols in flaxseed oil extracted by Soxhlet were determined by HPLC (LC‐6AD, Shimadzu, Tokyo, Japan) with a silica column (4.8 mm × 250 mm × 5 μm) in a two‐buffer gradient elution system at a flow rate of 0.8 ml/min: solvent A: B (99:1, v/v) at 0–13 min; A: B (100:0, v/v) at 13–24 min; A: B (4:96, v/v) at 24–30 min. The UV detector wavelength was set at 298 nm. The tocopherols were quantified by using α‐ and γ‐tocopherol standards, respectively. The contents of phytosterols were analyzed by Agilent 6,890 GC with a flame ionization detector and capillary DB‐5HT column (30.0 m × 220 μm × 0.10 μm) following derivation with 160 μl of Tri‐Sil at 105℃ for 15 min and redissolved in 1.0 ml of n‐hexane. The phytosterols were identified in comparison with authentic standards and quantified relative to the 5α‐Cholestane.

### 2.6 Analysis of crude fat content, fatty acid profiles, and triglyceride configuration in flaxseed

Freeze‐dried flaxseed sprouts were powdered and analyzed for crude fat content by continuous extraction in a Soxhlet apparatus for 12 hr using petroleum ether as solvent. The crude fat was expressed as follows: $X\left(\%\right)=\frac{m_{2}‐m_{1}}{m}*100\%,$ where *X* was the content of crude fat in samples; m was the mass of samples; m_1_ was the mass of extraction bottle; m_2_ was the mass of extraction bottle and crude fat. The main fatty acid (FA) profiles of flaxseed oil extracted by Soxhlet were determined using an Agilent 6890 GC equipped with a Flame Ionization Detector and a HP‐INNOWAX fused silica capillary column (30 m × 0.32 mm, 0.25 μm) as described by Deng et al., (2018).

The flaxseed oil was further dissolved into n‐hexane/isopropyl alcohol/acetonitrile (40:40:20, v/v/v) mixture at the concentration of 1.0 mg/ml and subjected to UPLC‐MS/MS systems for analysis of the TAG configuration (Xie et al., 2018) with slight modification. Briefly, the separation was performed on a LC‐30A UPLC instrument (Shimadzu, Tokyo, Japan) equipped with a Phenomenex Kinete C18 column (100 × 2.1 mm, 2.6 µm) in a two‐buffer gradient elution system at a flow rate of 0.4 ml/min: solvent A, H_2_O:MeOH:ACN (1:1:1, v/v/v) mixture with 5 mM of NH4Ac; solvent B, IPA:ACN (5:1, v/v) with 5 mM of NH4Ac; a gradient elution of 20% B at 0–0.5 min, 20%–40% B at 0.5–1.5 min, 40%–60% B at 1.5–3.0 min, 60%–98% B at 3.0–13.0 min, 98%–20% B at 13.0–13.1 min, 20% B at 13.1–17.0 min. A 4000 Q‐Trap mass spectrometer (AB Sciex, Toronto, Canada) in the electrospray ionization source operating in positive mode under selective reaction monitoring (SRM) scan was conducted. The collision energy was set at 25 eV, the declustering potential was 100 V, and the capillary voltage was set at 5.5 V. Both MS1 and MS2 quadrupoles were maintained at unit resolution. Quantification was conducted using the area under the mass spectral peak for individual SRM ion channels for each TAG molecules.

### 2.7 Analysis of antioxidant and oxidation stability of flaxseed and flaxseed oil

The 2, 2‐Diphenyl‐1‐picrylhydrazyl radical (DPPH•) scavenging activities and ferric reducing antioxidant powers (FRAP) of flaxseed sprouts and flaxseed oil extracted by Soxhlet were determined according to a previous method (Xiang et al., 2019). The results were expressed as milligram trolox equivalents (TE) and FeSO_4_ /100 g flaxseed sprouts (dry basis) or oil, respectively. The 2, 2′‐azinobis‐(3‐ethylbenzthiazoline 6‐sulfonic acid) radical cation (ABTS•+) scavenging activities of flaxseed sprouts and oil were conducted according to the commercial kit instructions (Beyotime Biotechnology Co., Ltd., Shanghai, China). The results were expressed as mg TE/100 g flaxseed sprouts (dry basis) or flaxseed oil.

The thermal stabilities, initial oxidation temperature (IOT) of flaxseed sprouts and flaxseed oil extracted by Soxhlet were determined by differential scanning calorimeter (Modulated DSC‐Q2000, TA Instruments, New Castle, America). Briefly, 8.0 mg of samples were sealed in an empty aluminum pan using an empty similar pan as reference. The samples were placed inside the calorimeter, kept at 30℃ for 2 min, and then heated up to 350℃ at the rate of 10℃/min in an oxygen flow of 30 ml/min. The IOT was the temperature at the inflection point where the absorption peak appeared in the scanning curve of the DSC instrument.

### 2.8 Statistical analysis

The results were presented as mean ±standard deviation (*n* = 3). Data were statistically analyzed using the SPSS statistical software (version 21.0, IBM, Chicago, IL, USA). Statistical analysis was conducted using one‐way analysis of variance (ANOVAs) and Duncan's multiple‐range test. A value of *p* <.05 was considered significant.

## 3 RESULTS AND DISCUSSION

### 3.1 Changes in morphology of flaxseed during germination

**FIGURE 1:**
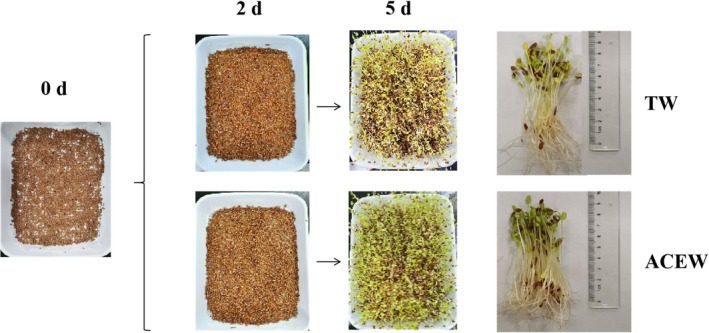
Changes in the morphology of flaxseed during germination. Abbreviations: ACEW, acidic electrolyzed water; TW, tap water

As depicted in Figure 1, the flaxseed began to germinate after 2 days of ACEW and TW treatments. The germination percentage of flaxseed treated with ACEW reached 97.45%, whicha increased by 18.25% when compared with that of TW (*p* <.05) following 5 days of treatment (seen in Figure S1), revealing a promotive effect on seed germination. The mean length of hypocotyls and radicles of flaxseed sprouts exposed to TW were 6.1 cm and 3.2 cm upon 5 days of germination, respectively. By contrast, the flaxseed sprouts increased by 35.56% in the length of radicles (*p* <.05), but not the hypocotyls when exposed to ACEW. Our findings were consistent with the previous study conducted by Liu et al., (2013), who found that ACEW was more favorable to the germination of brown rice due to its potential antibacterial activity.

### 3.2 Changes in polar lipid concomitants of flaxseed sprouts during germination

#### 3.2.1 Total phenolic and flavonoids

As shown in Table 1, the content of total phenolic acids in native flaxseed reached 97.89 mg/100 g, which was consistent with the result reported by Deng et al., (2018), but lower than the data found by Wang et al., (2015) due to the different flaxseed varieties. During 5 days of germination, the contents of total phenolic acids in flaxseed significantly and linearly increased, reaching the maximum value of 279.07 mg/100 g (*p* <.05). Although it showed a relatively small increment, the changing trend was in agreement with the previous findings obtained by Wang et al., (2015). The contents of total flavonoids in native flaxseed were 21.67 mg/100 g and increased by 2.73‐fold during 5 days of germination (*p* <.05), which was less than those reported by Wang et al., (2015) due to the different germinating conditions. Notably, ACEW further heightened the accumulation of total phenolic acids and flavonoids in flaxseed by 4.82% and 8.22% in comparison with TW after 5 days of germination (*p* <.05).

**TABLE 1:** Changes in the contents and composition of phenolic compounds in flaxseed during germination

Phenolic compounds	Germination treatment											
		Control	TW					ACEW				
		0	1	2	3	4	5	1	2	3	4	5
Total phenolic acids (mg/100 g)		97.89 ± 3.69a	121.40 ± 2.42b	146.42 ± 2.74d	163.29 ± 3.85e	190.63 ± 4.91g	279.07 ± 0.48i	135.32 ± 2.73c	160.96 ± 5.73e	179.64 ± 1.03f	202.64 ± 8.81h	292.52 ± 4.17j
Flavonoids (mg/100 g)		21.67 ± 0.13a	32.19 ± 0.08b	44.41 ± 0.88d	54.94 ± 0.24e	68.05 ± 0.61g	80.92 ± 0.33i	35.56 ± 0.21c	45.38 ± 0.35d	58.69 ± 0.96f	77.40 ± 0.45h	87.57 ± 0.57j
Alkaline hydrolysates of lignans	SDG (mg/g)	5.14 ± 0.16g	6.33 ± 0.11h	3.36 ± 0.06e	2.49 ± 0.13b	1.38 ± 0.08d	0.33 ± 0.04a	7.31 ± 0.08i	3.70 ± 0.17f	2.38 ± 0.13d	1.73 ± 0.13c	0.48 ± 0.04a
	FeAG (μg/g)	55.06 ± 2.25g	66.45 ± 1.85h	48.07 ± 1.73f	32.19 ± 1.59d	18.89 ± 0.61c	8.50 ± 0.44a	71.53 ± 1.54i	52.37 ± 1.73g	38.43 ± 1.23e	19.21 ± 0.79c	14.50 ± 0.98b
	*p*‐CouAG (μg/g)	80.93 ± 0.62e	97.54 ± 4.58f	48.93 ± 0.17d	39.09 ± 0.64c	33.62 ± 2.56bc	23.34 ± 1.26a	123.26 ± 5.83g	49.65 ± 3.32d	36.12 ± 1.76c	28.25 ± 1.75ab	25.85 ± 0.79a
	Protocatechuic acid	ND	ND	14.77 ± 1.12a	18.68 ± 0.35b	39.77 ± 1.30d	46.44 ± 0.16f	ND	16.49 ± 0.23ab	29.37 ± 1.75c	42.98 ± 2.06e	51.82 ± 1.97g
	4‐Hydroxybenzoic acid	21.60 ± 0.61a	21.87 ± 0.06a	20.67 ± 1.19a	21.88 ± 0.21a	23.56 ± 0.10b	37.59 ± 0.11e	24.85 ± 1.43b	24.10 ± 0.06b	26.14 ± 0.17c	27.66 ± 0.75d	43.07 ± 0.79f
Free phenolic acids (µg/g)	Syringic acid	4.21 ± 0.24bc	4.57 ± 0.01c	3.76 ± 0.28a	4.00 ± 0.30ab	5.08 ± 0.06d	4.54 ± 0.01e	3.62 ± 0.13a	3.78 ± 0.03a	5.08 ± 0.17d	5.59 ± 0.21e	4.37 ± 0.20bc
	*p*‐Coumaric acid	4.19 ± 0.16a	13.01 ± 0.64b	17.03 ± 0.48c	21.22 ± 1.39d	27.01 ± 1.442e	33.04 ± 0.01f	13.66 ± 0.37b	18.04 ± 0.07c	27.38 ± 0.96e	35.76 ± 1.77g	44.05 ± 1.90h
	Ferulic acid	31.63 ± 0.59a	37.26 ± 1.47ab	42.02 ± 1.32bc	86.66 ± 2.15d	114.98 ± 3.76e	136.04 ± 5.30g	39.79 ± 0.83bc	45.00 ± 1.24c	89.33 ± 0.83d	124.08 ± 6.29f	192.29 ± 2.60h
	Caffeic acid	3.43 ± 0.18a	5.33 ± 0.18b	6.25 ± 0.20c	5.35 ± 0.16b	7.09 ± 0.07d	8.57 ± 0.20f	6.01 ± 0.14c	6.37 ± 0.01c	7.10 ± 0.30d	7.70 ± 0.18e	9.13 ± 0.24g
	Vanillin	2.91 ± 0.14a	3.99 ± 0.17c	3.36 ± 0.13b	3.35 ± 0.10b	3.99 ± 0.16c	4.42 ± 0.07d	3.24 ± 0.11b	3.40 ± 0.03b	4.40 ± 0.17d	4.78 ± 0.18e	4.84 ± 0.16e
	Gallic acid	16.97 ± 0.18a	36.47 ± 0.01b	72.66 ± 3.38d	88.26 ± 5.83e	92.13 ± 2.98ef	102.52 ± 3.66g	44.86 ± 1.02c	99.36 ± 1.43fg	101.64 ± 2.22g	112.23 ± 3.93h	127.89 ± 6.86i

Note

ND:not detected; The means with different letters are significantly different at *p* <.05 level.

Abbreviations: ACEW, acidic electrolyzed water; TW, tap water;SDG, secoisolariciresinol diglucoside; FeAG, ferulic acid glucoside; p‐CouAG, p‐coumaric glucoside

**FIGURE 2:**
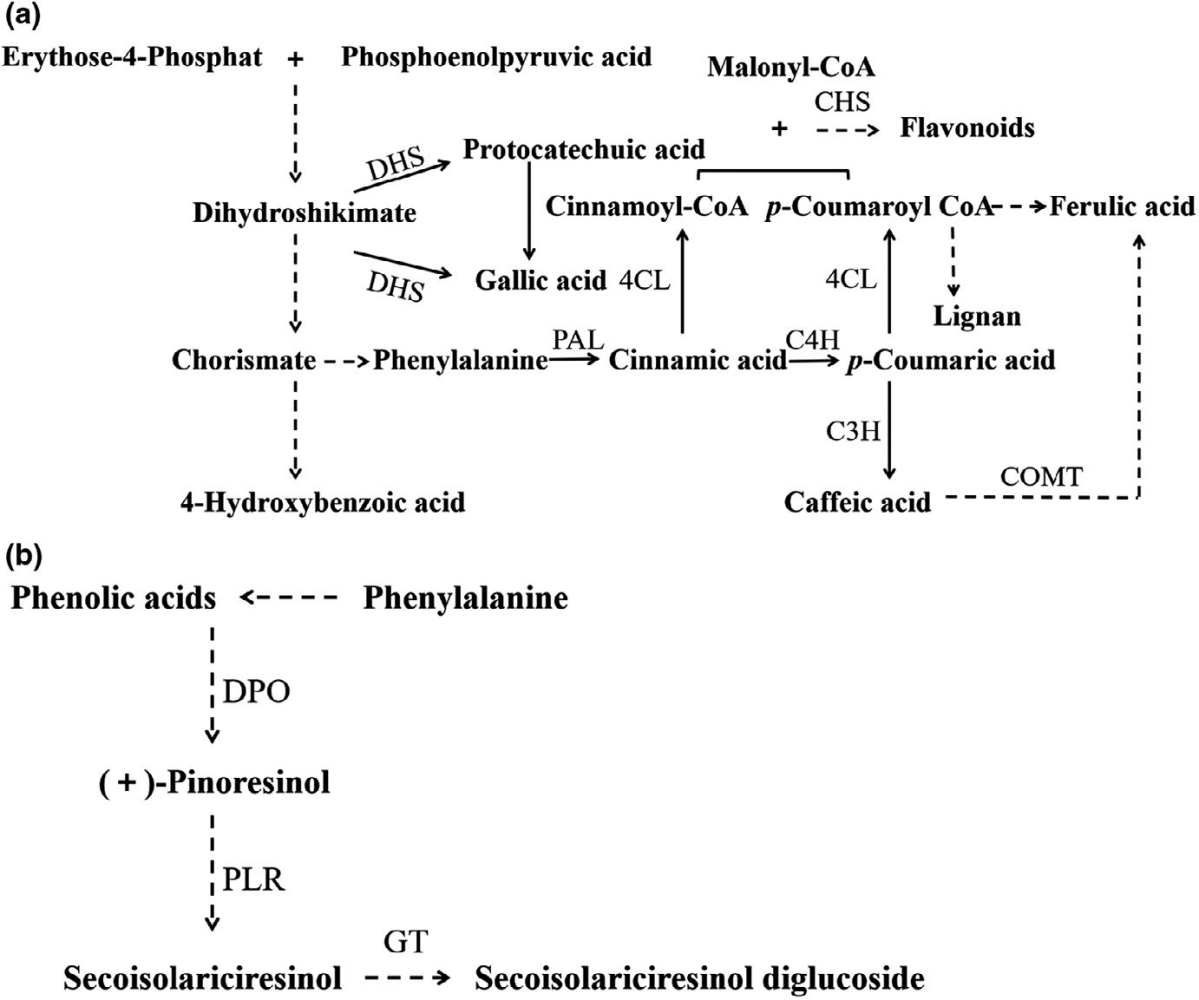
The possible biosynthesis pathway of phenolic acids (a) and lignan secoisolariciresinol diglucoside (b) in flaxseed during germination. The dashed line represents the omission of the synthesis step, and the solid line represents the direct synthesis. Abbreviations: 4CL, 4‐coumarate CoA ligase; C3H, p‐coumarate 3‐hydroxylase; C4H, cinnamate 4‐hydroxylase; CHS, chalcone synthase; COMT, caffeic acid o‐methyltransferase; DHS, dehydroshikimate dehydratase; DPO, dirigent‐protein oxidase; GT, glucosyltransferase; PAL, phenylalanine ammonia lyase; PLR, pinoresinol‐lariciresinol reductase

The accumulation of phenolic acids could be explained by the activation of key enzymes or cofactors responsible for seed germination (Wang et al., 2016). As depicted in Figure 2(a), the phenylpropanoid metabolic pathway might be induced by phenylalanine ammonia lyase (PAL) during flaxseed germination, a main pathway for phenolic acid synthesis. The intermediates of glycolysis and pentose phosphate pathways led to the generation of phenylalanine via shikimic acid pathway, which was accompanied by the continuous modification and release of multiple phenolic acids. In fact, the flavonoid synthesis was also simultaneously activated by chalcone synthase owing to the production of intermediate coumaroyl‐CoA and malonyl‐CoA (Chen et al., 2019; Vogt, 2010).

As shown in Table 1, the predominant free phenolic acids in native flaxseed were 4‐hydroxybenzoic acid, syringic acid, *p*‐coumaric acid, ferulic acid, caffeic acid, vanillin, and gallic acid, accounting for 25.42%, 4.96%, 4.93%, 37.23%, 4.04%, 3.43%, and 19.98% of the total amount of phenolic acids detected (84.94 μg/g), respectively. The inconsistent abundance and profiles of free phenolic acids in flaxseed were reported by Deng et al., (2018), which might be explained by the different planting area, harvest year, and seed maturity. The amounts of free phenolic acids in flaxseed increased by 2.68‐fold following 5 days of germination treated with TW (*p* <.05). Moreover, the increment in individual free phenolic acid was differently induced with an increase by 74.02%, 6.89‐fold, 3.30‐fold, 1.50‐fold, and 5.04‐fold for 4‐hydroxybenzoic acid, *p*‐coumaric acid, ferulic acid, caffeic acid, and gallic acid, respectively (*p* <.05). Surprisingly, the protocatechuic acid was synthesized after 2 days of flaxseed germination with the value of 14.77 μg/g and then increased by 2.14‐fold after 5 days of germination (*p* <.05). A further ascension of free phenolic acids by 27.83% had been displayed for flaxseed treated with ACEW when compared to those of TW (*p* <.05). Notably, the ferulic acid and *p*‐coumaric acid were specifically accumulated in flaxseed with the increment of 41.35% and 33.32%, respectively (*p* <.05).

As previously reported, the gallic acid could be directly formed after the dehydration of shikimic acid, and the 4‐hydroxybenzoic acid could be synthesized by chorismate under the catalysis of isochorismate and pyruvate lyase (Vogt, 2010). The *p*‐coumaric acid was generated under the action of PAL and cinnamate 4‐hydroxylase (C4H) and was further formed caffeic acid and *p*‐coumaroyl‐CoA, which could further converted into lignan secoisolariciresinol diglucoside, flavonoids, and ferulic acid (Vogt, 2010). Moreover, the syringic acid and vanillin content in flaxseed displayed no obvious change, which might be the biosynthesis and conversion depletion of syringic acid and vanillin were in dynamic equilibrium. The relatively lagging synthesis of protocatechuic acid might be due to the activation of 3‐dehydroshikimate dehydratase via the shikimate synthesis pathway (Li et al., 1999). However, the specific enrichments of ferulic acid and *p*‐coumaric acid in flaxseed were thanks to the further activation of the PAL and C4H enzyme activities by ACEW.

#### 3.2.2 Flaxseed lignans

As seen in Table 1, the contents of SDG, FeAG, and *p*‐CouAG in native flaxseed were 5.25 mg/g, 55.06 μg/g, and 80.93 μg/g, respectively, which were relatively lower than that reported by Deng et al., (2018). The SDG content in flaxseed increased by 21.90% after 1 day of germination (*p* <.05), and then dropped by 93.33% following 2–5 days of germination by TW (*p* <.05). Similar changing trends had been observed for *p*‐CouAG and FeAG in flaxseed treated with TW following 5 days of germination. This tendency was consistent with the results reported by Li et al., (2019). However, Wang et al., (2016) had observed that the SDG content in flaxseed continuously increased during 0–8 days of germination and then decreased when the germination time extended for 10 days. Notably, the expression patterns of the dirigentprotein oxidase (DPO) and glucosyl transferase (GT) responsible for SDG synthesis might be inconsistent with our research and Li et al., (2019), due to the different flaxseed varieties and water stress condition. By contrast, the amounts of SDG, FeAG, and *p*‐CouAG in flaxseed upon 1 day of ACEW treatment were 15.48%, 7.64%, and 26.37% higher than those of TW treatment (*p* <.05). According to Wang et al., (2016), a possible biosynthetic pathways of lignans were depicted in Figure 2(b). The key genes for SDG synthesis might be further activated by ACEW, including the DPO and GT (Toure & Xu, 2010; Wang et al., 2016). In addition, the increase in content of *p*‐CouAG might becaused by the initiation of glycosylation of *p*‐coumaric acid induced by ACEW. However, the glucoside of ferulic acid might be blocked when compared with the *p*‐CouA, which may be caused by the competitive glycosidation between ferulic acid and *p*‐CouAG. The incoordinated increase between the SDG, *p*‐CouAG, and FeAG might lead to a decrease in molecular weight of the lignans macromolecule and further improve antioxidant activity and migration characteristics, owing to the cross‐linkage of *p*‐CouAG and FAG onto the SDG glycosides (Ramsay et al., 2017).

#### 3.2.3 Cyclolinopeptides

**FIGURE 3:**
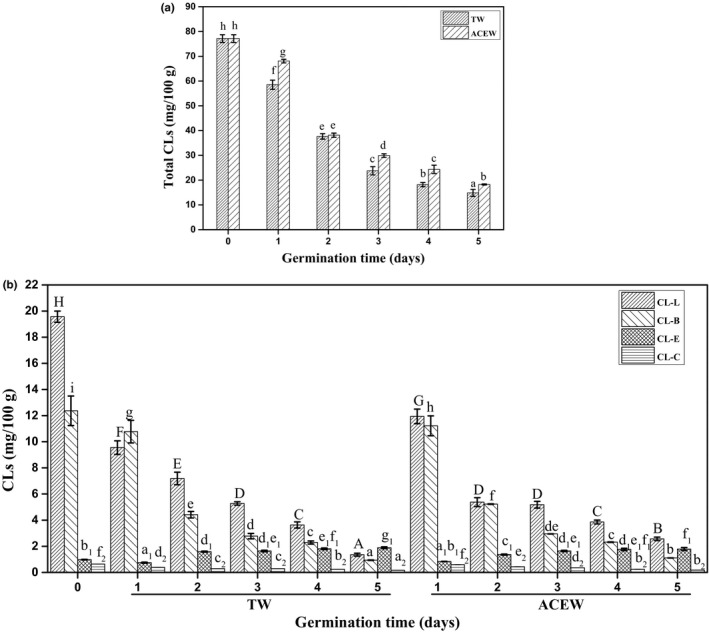
Changes in the contents of total CLs (a) and CL‐B, E, C, L (b) in flaxseed during germination. Abbreviations: ACEW, acidic electrolyzed water; CL, cyclolinopeptide; TW, tap water. The means with different letters are significantly different at p <.05 level

The CLs are hydrophobic cyclic peptides mainly existed in cotyledons of flaxseed. As shown in Figure 3 and Table S1, eight CLs were identified in native flaxseed with 77.14 mg/100 g of total CLs, including CL‐O (25.65%), CL‐L (25.37%), CL‐A (16.92%), CL‐B (16.03%), CL‐*M* (7.9%), CL‐*N* (6.0%), CL‐E (1.3%), and CL‐C (0.8%), respectively. Similar species but different proportion of CLs in flaxseed were reported by Lao et al., (2014), which might be attributed to the different flaxseed varieties. The amounts of total CLs in flaxseed decreased by 80.76% following 5 days of germination by TW (*p* <.05), which might be explained by the gradual disappearance of cotyledons when the flaxseed developed into leaves (Gui et al., 2012). Moreover, the change trends of individual CL in flaxseed were differently affected during germination, with a decrease by 73.44%, 92.40%, 69.73%, 87.94%, 81.61%, 91.30%, and 75.49% for CL‐C, CL‐B, CL‐A, CL‐L, CL‐O, CL‐N, and CL‐M, respectively (*p* <.05), whereas the content of CL‐E in flaxseed increased by 48.15% (*p* <.05), which might be due to the intermolecular conversion from CL‐L (Lao et al., 2014). As previously reported, the CL‐C, from the preferential oxidation of sulfhydryl group of CL‐B, could be further oxidized into the CLs with methionine S, S‐dioxide, thus leading to the synchronous reduction of CL‐B and CL‐C (Jadhav et al., 2013; Lao et al., 2014). The above oxidation–reduction reaction among CLs was easily occurred during the process of energy supply, just like the flaxseed germination (Lao et al., 2014). Compared to the TW, a more moderate downward trend in total CLs had been displayed for the flaxseed treated with ACEW. Simultaneously, the ACEW‐mediated reduction of individual CL‐C, CL‐B, CL‐A, CL‐L, CL‐O, CL‐N, and CL‐M was almost in the same proportion, ranging from 67.75% to 91.03%. However, the content in CL‐E was relatively decreased than that by TW (*p* <.05), suggesting that ACEW could improve the bitterness of flaxseed sprouts relativel to TW.

### 3.3 Changes in nonpolar lipid concomitants of flaxseed sprouts during germination

#### 3.3.1 Tocopherols

**FIGURE 4:**
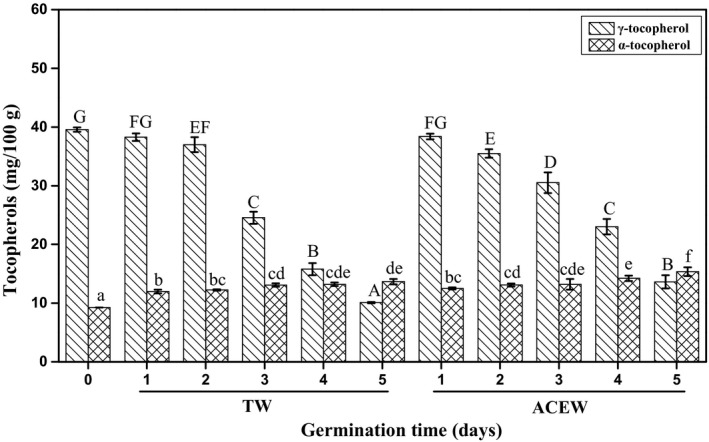
Changes in the contents of α‐ and γ‐tocopherol of flaxseed during germination. Abbreviations: ACEW, acidic electrolyzed water; TW, tap water. The means with different letters are significantly different at p <.05 level

Tocopherols, including α‐, γ‐, β‐, and δ‐tocopherol isomers, are mainly distributed on the membrane phospholipids of oil bodies in oilseeds and exert the in situ antioxidant activity against specific environmental stress (Munoz & Munne‐Bosch, 2019). As shown in Figure 4, the content of total tocopherols in native flaxseed reached 48.81 mg/100 g oil, mainly existed in γ‐tocopherol isomer (81.01%), followed by α‐tocopherol isomer (18.99%), which was in accordance with the results conducted by Choo et al., (2007). A weak increase in total tocopherols in flaxseed had been exhibited after 1 day of germination and then substantially decreased with the lowest value of 23.52 mg/100 g oil after 5 days of germination. In particular, the individual tocopherols in flaxseed differently varied during 5 days of germination. In which, the α‐tocopherol content in flaxseed increased by 42.07%, whereas the content of γ‐tocopherol decreased by 74.49% (*p* <.05), which was similar to findings reported by Li et al., (2019). However, ACEW led to a further accumulation of α‐tocopherol (14.35 mg/100 g oil), which was accompanied by the slower decrease in γ‐tocopherol following 5 days of germination (*p* <.05) in comparison with that of TW. Potentially, the γ‐tocopherol could be converted into α‐tocopherol under the action of γ‐tocopherol methyltransferase via α‐tocopherol biosynthetic pathway (Shintani & DellaPenna, 1998), which might be further activated by ACEW during germination. According to the different antioxidant potential of tocopherol isomers, the conversion from γ‐tocopherol to α‐tocopherol isomers, as well as the endogenous synthesis of α‐tocopherol isomer in flaxseed treated with ACEW, were beneficial to enhance the oxidation stability of flaxseed (Lars et al., 2010).

#### 3.3.2 Phytosterols

**FIGURE 5:**
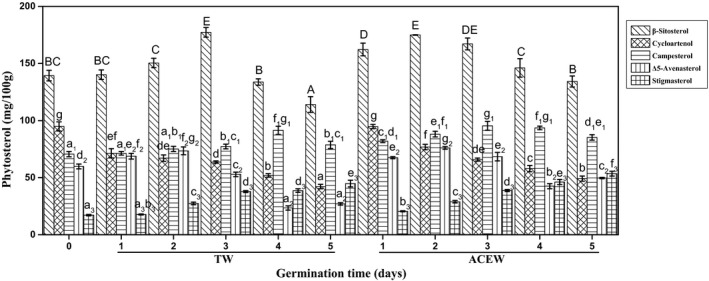
Changes in the contents and composition of phytosterols in flaxseed during germination. Abbreviations: ACEW, acidic electrolyzed water; TW, tap water. The means with different letters are significantly different at p <.05 level

Phytosterols are one of the key substances of plant cell membrane as scaffolds (Moreau et al., 2018). As shown in Figure 5, the content of total phytosterols in native flaxseed achieved 381.74 mg/100 g oil, which was in agreement with the results from Herchi et al., (2009). Similar to Deng et al., (2018), the *β*‐sitosterol, cycloartenol, campesterol, Δ5‐oatosterol, and stigmasterol were identified, accounting for 36.50%, 24.85%, 18.49%, 15.67%, and 4.49% of total phytosterols, respectively. The contents of total phytosterols increased by 13.89% after 3 days of germination (*p* <.05) and then declined by 14.59% when the germination time further extended to 5 days (*p* <.05). The above results were similar to those observed in germinated soybeans (Shi et al., 2010). In which, the contents of *β*‐sitosterol, campesterol, and Δ5‐avenasterol in flaxseed behaved firstly rising and then decreasing trend, reaching the maximum values of 177.23 mg/100 g, 94.74 mg/100 g, and 76.08 mg/100 g after 2–3 days of germination, respectively. By contrast, the content of cycloartenol in flaxseed decreased by 55.59% (*p* <.05), whereas the level of stigmasterol increased by 1.62‐fold (*p* <.05) during 5 days of germination. In comparison, the ACEW accelerated the accumulation of phytosterols in flaxseed, especially for *β*‐sitosterol, reaching the highest value similar to that of TW after 1–2 days of germination. Previous study had reported that the *β*‐sitosterol and campestanol could be converted into stigmasterol under the action of 22‐desaturase enzymes during flaxseed development, which might contribute to the accumulation of stigmasterol in flaxseed sprouts during the early germination (Herchi et al., 2009). However, the decreasing content of cycloartenol in flaxseed sprouts might be explained by the conversion of cycloartenol to 24‐methylene cycloartanol catalyzed by S‐adenosylmethionine‐cycloartenol‐C24‐ methyltransferase during the metabolism of phytosterols.

### 3.4 Changes in lipid content, fatty acid profiles, and triglyceride configuration of flaxseed sprouts during germination

The lipids exist in form of oil bodies in oilseed, which provides energy for seed germination as a high‐energy carbon reserve, and protected the lipid oxidation against various environmental stress (Nikiforidis, 2019). As shown in Table 2, the total lipid content of native flaxseed was 39.64%, which belonged to the ranges reported by Deng et al., (2018). As expected, no obvious changes in total lipid content had been observed for flaxseed treated with TW during 2 days of germination and then declined by 45.51% upon 5 days of germination (*p* <.05). Our results were in line with those reported by Herchi et al., (2015). As previously reported (Wanasundara et al., 1999), the lipase activity in flaxseed might be initially upregulated and then reached a plateau after 2 days of germination. By contrast, the total fat content of flaxseed treated with ACEW started to decrease after 3 day of germination and then dropped by 41.32% with the lowest value of 23.26% (*p* <.05). The ACEW might reduce the number of microorganisms, which shared the energy required for the growth of flaxseed sprouts (Liu et al., 2013). In particular, ACEW led to the preferential depletion of carbohydrates as demonstrated by the lower total sugar contents in flaxseed (Seen in Table S2). As presented in Table 2, the α‐linolenic acid (ALA/C18:3n‐3), oleic acid (OA/C18:n‐9), linoleic acid (LA/C18:2n‐6), palmitic acid (PA/C16:0), and stearic acid (SA/C18:0) were identified in native flaxseed oil extracted by Soxhlet, accounting for 54.03%, 22.96%, 12.59%, 5.90%, and 4.52%, respectively. The proportions of ALA and PA in flaxseed oil extracted by Soxhlet decreased by 1.5% (*p* <.05) and 0.40%, which was parallel with the ascending proportions of 1.18% (*p* <.05) and 0.55% for LA and SA during 5 days of germination.

**TABLE 2:** Changes in the fatty acid profiles of flaxseed during germination

FA composition (%)	Germination treatment										
	Control	TW					ACEW				
	0	1	2	3	4	5	1	2	3	4	5
C16:0	5.90 ± 0.02de	5.91 ± 0.07de	5.78 ± 0.02de	5.56 ± 0.04de	5.36 ± 0.04bc	5.43 ± 0.04a	5.96 + 0.09e	5.76 ± 0.10 cd	5.73 ± 0.008 cd	5.54 ± 0.02ab	5.53 ± 0.06ab
C18:0	4.52 ± 0.04b	4.40 ± 0.04a	4.56 ± 0.03b	4.83 ± 0.01d	5.10 ± 0.01f	5.04 ± 0.02f	4.53 ± 0.004b	4.69 ± 0.09c	4.70 ± 0.03c	4.93 ± 0.01e	4.85 ± 0.04de
C18:1*n*−9	22.96 ± 0.16ab	22.91 ± 0.14a	22.86 ± 0.12a	23.04 ± 0.12abc	23.30 ± 0.01c	23.03 ± 0.08abc	23.02 ± 0.10abc	23.27 ± 0.04bc	23.01 ± 0.21abc	23.09 ± 0.09abc	23.02 ± 0.18abc
C18:2n−6	12.59 ± 0.02a	12.86 ± 0.05b	12.96 ± 0.12bc	13.15 ± 0.15de	13.64 ± 0.05f	13.97 ± 0.04a	12.67 ± 0.01a	12.97 ± 0.05bc	13.03 ± 0.02 cd	13.23 ± 0.07e	13.29 ± 0.01e
C18:3n−3	54.03 ± 0.28e	53.92 ± 0.10e	53.85 ± 0.16e	52.42 ± 0.24d	50.61 ± 0.45c	47.52 ± 0.61a	53.81 ± 0.04e	53.32 ± 0.24e	53.53 ± 0.31e	51.21 ± 0.27c	48.30 ± 0.24b

Note

Abbreviations: C16:0: palmitic acid; C18:0: stearic acid; C18:2n‐6: linoleic acid; C18:3n‐3: α‐linolenic acid; C18:1*n*‐9: oleic acid; FA: fatty acid.

The means with different letters are significantly different at *p* <.05 level.

The degradation of FAs, especially for ALA, was specifically related to its Sn‐1/2/3 position in glycerol skeleton in flaxseed. As shown in Table 3, the ALA, OA, LA, PA, and SA in native flaxseed were mainly located in the Sn‐2/3, Sn‐1/2, Sn‐ 2/3, Sn‐1, and Sn‐1 of glycerol skeleton, accounting for 89.12%, 63.26%, 28.36%, 16.09%, and 25.58%, respectively. Especially, the ALA on Sn‐2/3 was generally co‐existed with LA and OA on Sn‐1 of glycerol skeleton, such as 1‐oleic acid‐2,3‐linolenic acid glyceride (OLnLn) for 17.46% and 1‐linoleic acid‐2,3‐linolenic acid glyceride (LLnLn) for 9.81%. After 5 days of germination treated with TW, the proportion of ALA in Sn‐1/2/3 (1,2,3‐linolenic acid glyceride, LnLnLn) and Sn‐2/3 (OLnLn) declined by 5.11% and 1.89% (*p* <.05), respectively, which was accompanied by the increase in ALA on Sn‐3 (1,2‐oleic acid‐3‐linolenic acid glyceride, OOLn) and Sn‐2/3 (LLnLn) of glycerol skeleton (*p* <.05). The above results suggested that the ALA on Sn‐2 was most easily depleted when the Sn‐1 of glycerol skeleton was substituted for OA in flaxseed treated with TW. By contrast, the content of ALA in Sn‐1/2/3 (LnLnLn) of flaxseed decreased by 2.56% (*p* <.05), whereas the ALA in Sn‐2/3 (LLnLn) increased by 6.22% (*p* <.05) following 5 days of ACEW exposure. The relatively inhibiting effect of ACEW on the preferential metabolism of ALA on Sn‐2/3 (OLnLn) had been obtained. ALA might be preferentially degraded by lipase and subsequently participated in the tricarboxylic acid cycle during germination, which was inhibited by ACEW, and thus was more conducive to retain ALA in flaxseed during germination.

**TABLE 3:** Changes in the triglyceride configuration of flaxseed during germination

Type	m/z: Da	TAGs structure	Relative percentage (%)				
			Control 0 d	TW 3 d	TW 5 d	ACEW 3 d	ACEW 5 d
[M+NH4]+	894.75	OLnLn	17.46	17.33	17.13	17.41	17.24
[M+NH4]+	890.72	LnLnLn	15.26	14.78	14.48	15.06	14.87
[M+NH4]+	896.77	OLLn SLnLn	12.13	12.28	12.38	12.03	12.01
[M+NH4]+	898.79	OOLn	12.11	12.36	12.52	12.54	12.87
[M+NH4]+	892.74	LLnLn	9.81	9.98	10.69	10.12	10.42
[M+NH4]+	900.80	SOLn OOL	6.70	6.74	6.99	6.85	6.95
[M+NH4]+	868.74	PLnLn	5.16	5.05	4.82	5.16	5.14
[M+NH4]+	902.82	OOO SOL	4.88	4.92	5.17	4.97	5.01
[M+NH4]+	872.77	POLn	4.30	4.21	4.17	4.28	4.31
[M+NH4]+	870.75	PLLn	2.50	2.56	2.70	2.48	2.53
[M+NH4]+	876.80	POO	2.16	2.16	2.14	2.22	2.34
[M+NH4]+	874.79	POL PSLn	1.97	1.96	1.97	1.97	1.99
[M+NH4]+	904.83	SOO	1.87	1.92	2.01	1.86	1.82

Abbreviations: L: linoleic acid; Ln: α‐linolenic acid; O: oleic acid; P: palmitic acid; S: stearic acid; TAG: triglyceride.

### 3.5 Changes in the oxidative and antioxidant properties of flaxseed and flaxseed oil

**FIGURE 6:**
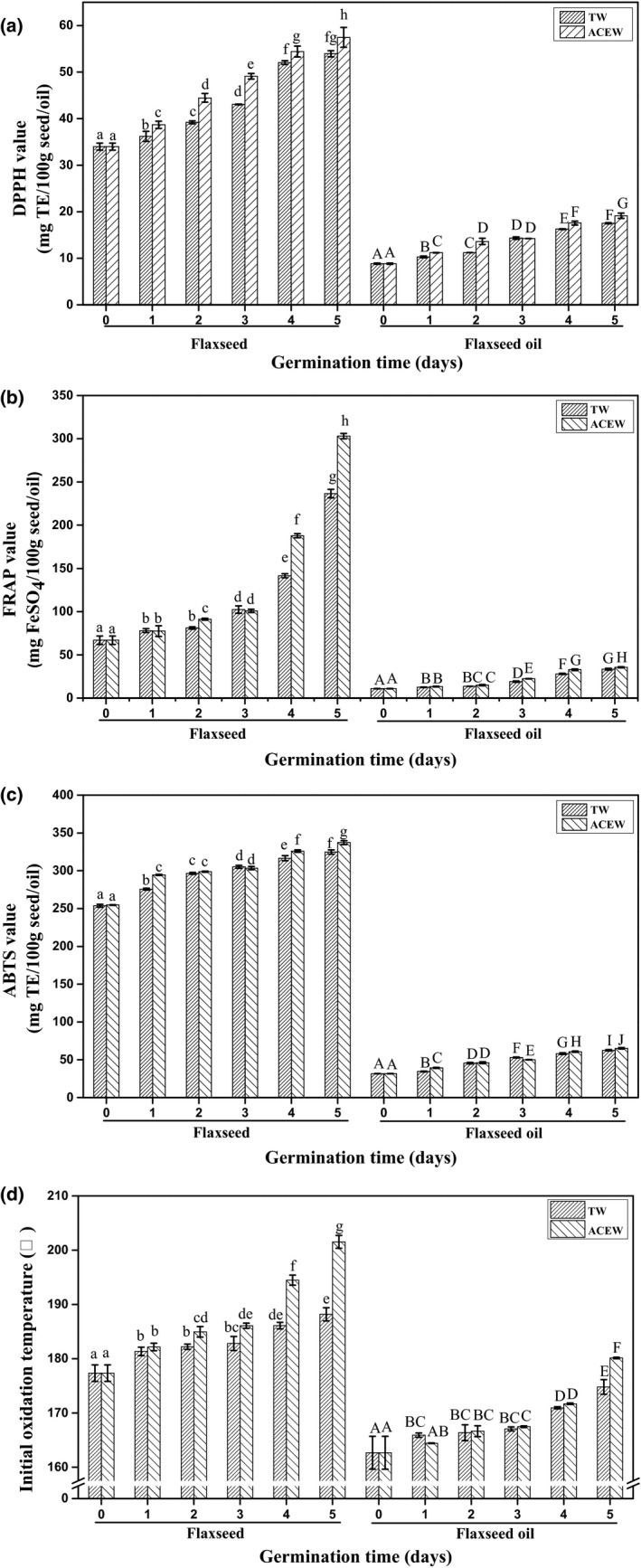
Changes in the DPPH (a), FRAP (b), ABTS (c) values, and initial oxidation temperature (d) of flaxseed and flaxseed oil during germination. Abbreviations: ACEW, acidic electrolyzed water; TW, tap water; TE, trolox equivalents . The means with different letters are significantly different at p <.05 level

As depicted in Figure 6(a)–(c), the values of DPPH, FRAP, and ABTS in native flaxseed were 34.51 mg TE/100 g, 67.00 mg FeSO_4_/100 g, and 254.96 mg TE/100 g, respectively. After 5 days of germination, the values of DPPH, FRAP, and ABTS in flaxseed increased by 57.61%, 2.48‐fold, and 26.58%, respectively (*p* <.05), which was in line with the data from Li et al., (2019), who found the free radicals‐scavenging abilities of DPPH and ABTS were 26.59%–64.80% and 31.76%–48.93%, respectively. As expected, ACEW further raised the values of DPPH, FRAP, and ABTS in flaxseed by 6.55%, 28.07%, and 3.86% following 5 days of germination (*p* <.05). Consistent with the findings from Barthet et al., (2014), the values of DPPH, FRAP, and ABTS of native flaxseed oil extracted by Soxhlet were 8.95 mg/100 g oil, 11.41 mg/100 g oil, and 31.50 mg/100 g oil, respectively, which increased by 94.86%, 1.93‐fold, and 96.60% during 5 days of germination (*p* <.05). The values of DPPH, FRAP, and ABTS in flaxseed oil were further increased by 9.18%, 6.53%, and 4.21% by ACEW treatment, when compared to those of TW after 5 days of germination (*p* <.05).

The initial oxidation temperature (IOT) is an important indicator for evaluating the thermal oxidation stability of flaxseed and flaxseed oil. A superior thermal oxidation stability was accompanied by a higher IOT value. As exhibited in Figure 6(d), the IOT values of native flaxseed and flaxseed oil extracted by Soxhlet were 178.42℃ and 162.27℃, respectively, which ascended by 5.94% and 7.45% after 5 days of germination when treated with TW (*p* <.05). Comparatively, higher values of IOT had been observed for flaxseed and flaxseed oil during 5 days of germination by ACEW, reaching 200.68℃ and 180.13℃, respectively (*p* <.05).

### 3.6 The correlation analysis and principal component analysis

As shown in Table 4, the DPPH value of flaxseed showed significantly positive correlation to ABTS and FRAP values (for ABTS, *r* = 0.905, *p* <.01; for FRAP, *r* = 0.929, *p* <.01). During 0–1 day of germination, a strong correlation had been observed between DPPH values and total phenolic acids (mainly for caffeic acid, ferulic acid, *p*‐coumaric acid, gallic acid, vanillin, but not 4‐hydroxybenzoic acid, syringic acid, protocatechuic acid), flavonoids, and lignans (for total phenolic acids, *r =* 0.986, *p* <.05; for flavonoids, *r* = 0.984, *p* <.05; for lignans, *r =* 0.992, *p* <.01). A direct correlation was obtained between the SDG and total phenolic acids (*r =* 0.957, *p* <.05), which indicated that the lignans and phenolic acids cooperatively constituted the material basis of endogenous antioxidant system in flaxseed during early germination. Due to the decrease in lignans contents with the extending germination time (2‐5 days), the DPPH values were merely positively related to total phenolic acids (primarily for 4‐hydroxybenzoic acid, *p*‐coumaric acid, caffeic acid, protocatechuic acid, ferulic acid, gallic acid, but not syringic acid, vanillin) (*r =* 0.909; *p* <.01) and flavonoids (*r =* 0.978; *p* <.01). These results suggested that the abundances and profiles of lipid concomitants for constituting the endogenous antioxidant system of flaxseed dynamically changed during different germination periods.

**TABLE 4:** The correlation analysis between the thermal oxidation stability and antioxidant capacities and lipid concomitants of flaxseed during germination

		Germination (0–1 day)				Germination (1–5 days)			
		DPPH (mg/100 g)	FRAP (mg/100 g)	ABTS (mg/100 g)	IOT (°C)	DPPH (mg/100 g)	FRAP (mg/100 g)	ABTS (mg/100 g)	IOT (°C)
Polar lipid concomitants	Total phenolic acids (mg/100 g)	0.986^b^	0.999^a^	0.999^a^	0.970^b^	0.909^a^	0.993^a^	0.845^a^	0.975^a^
	Flavonoids (mg/100 g)	0.984^b^	0.999^a^	0.999^a^	0.968^b^	0.978^a^	0.919^a^	0.965^a^	0.951^a^
	Total CLs (mg /100 g)	−0.978^b^	−0.998^a^	−0.998^a^	−0.972^b^	−0.874^a^	−0.735^b^	−0.977^a^	−0.785^a^
	SDG (mg/g)	0.992^a^	0.964^b^	0.966^b^	0.897^b^	−0.889^a^	−0.799^a^	−0.984^a^	−0.825^a^
	*p*‐CouAG (μg/g)	0.984^b^	0.999^a^	0.999^a^	0.972^b^	−0.762^b^	−0.656^b^	−0.944^a^	−0.686^b^
	FeAG (μg/g)	0.982^b^	0.999^a^	0.999^a^	0.969^b^	−0.907^a^	−0.822^a^	−0.993^a^	−0.854^a^
	protocatechuic acid (μg/g)	‐	‐	‐	‐	0.893^a^	0.974^a^	0.938^a^	0.982^a^
	4‐hydroxybenzoic acid (μg/g)	0.372	0.225	0.234	0.767^a^	0.962^a^	0.622	0.909^a^	0.767^a^
	syringic acid (μg/g)	0.536	0.421	0.421	0.749^b^	0.638	0.515	0.705^b^	0.749^b^
	*p*‐coumaric acid (μg/g)	0.988^b^	0.992^a^	0.992^a^	0.983^a^	0.944^a^	0.943^a^	0.972^a^	0.983^a^
	caffeic acid (μg/g)	0.993^a^	0.997^a^	0.999^a^	0.979^a^	0.883^a^	0.918^a^	0.934^a^	0.979^a^
	ferulic acid (μg/g)	0.999^a^	0.985^b^	0.989^b^	0.876^a^	0.943^a^	0.804^a^	0.937^a^	0.876^a^
	vanillin (μg/g)	0.976^b^	0.929	0.934	0.512	0.344	0.282	0.474	0.512
	gallic acid (μg/g)	0.988^b^	0.999^a^	0.995^a^	0.841^a^	0.734^b^	0.970^a^	0.770^a^	0.841^a^
	campesterol (mg/100 g)	0.66	0.525	0.541	0.478	0.372	0.036	0.507	0.164
Nonpolar lipid concomitants	stigmasterol (mg/100 g)	0.84	0.747	0.767	0.785^b^	0.892^a^	0.774^a^	0.856^a^	0.833^a^
	*β*‐sitosterol (mg/100 g)	0.527	0.377	0.395	0.330	−0.428	−0.479	−0.197	−0.480
	cycloartenol (mg/100 g)	0.996^a^	0.993^a^	0.996^a^	0.970^b^	−0.886^a^	−0.689^b^	−0.721^b^	−0.784^a^
	Δ5‐avenasterol (mg/100 g)	−0.942	−0.985^b^	−0.982^b^	−0.957^b^	−0.940^a^	−0.927^a^	−0.834^a^	−0.936^a^
	γ‐tocopherol (mg/100 g)	−0.854	−0.929	−0.920	−0.890	−0.981^a^	−0.880^a^	−0.922^a^	−0.932^a^
	α‐tocopherol (mg/100 g)	0.997^a^	0.993^a^	0.994^a^	0.942^b^	0.848^a^	0.660^b^	0.900^a^	0.747^b^

Abbreviations: CL: cyclolinopeptide; SDG: secoisolariciesinol diglucoside ; p‐CouAG: p‐coumaric glucoside

^a^ Correlation is significant at the 0.01 level.

^b^ Correlation is significant at the 0.05 level.

A positive correlation was obtained between the IOT and total phenolic acids (*r =* 0.970; *p* <.01), flavonoids (*r =* 0.968; *p* <.01), SDG (*r =* 0.897; *p* <.05), stigmasterol (*r =* 0.970; *p* <.05), α‐tocopherol (*r =* 0.942; *p* <.05), 4‐hydroxybenzoic acid (*r =* 0.767; *p* <.01), syringic acid (*r =* 0.749; *p* <.05), *p*‐coumaric acid (*r =* 0.983; *p* <.01), ferulic acid (*r =* 0.979; *p* <.01), and caffeic acid (*r =* 0.841; *p* <.01) during 0–1 day of germination. However, with the germination time further extended, a negative correlation was obtained between the IOT and SDG (*r =* −0.825; *p* <.05), which was accompanied by a position correlation with total phenolic acids (*r =* 0.975; *p* <.01), flavonoids (*r =* 0.951; *p* <.01), stigmasterol (*r =* 0.833; *p* <.05), α‐tocopherol (*r =* 0.747; *p* <.05), protocatechuic acid (*r =* 0.982; *p* <.01), 4‐hydroxybenzoic acid (*r =* 0.767; *p* <.01), *p*‐coumaric acid (*r =* 0.944; *p* <.01), ferulic acid (*r =* 0.883; *p* <.01), caffeic acid (*r =* 0.943; *p* <.01), and gallic acid (*r =* 0.734; *p* <.05), similar to antioxidant system.

**FIGURE 7:**
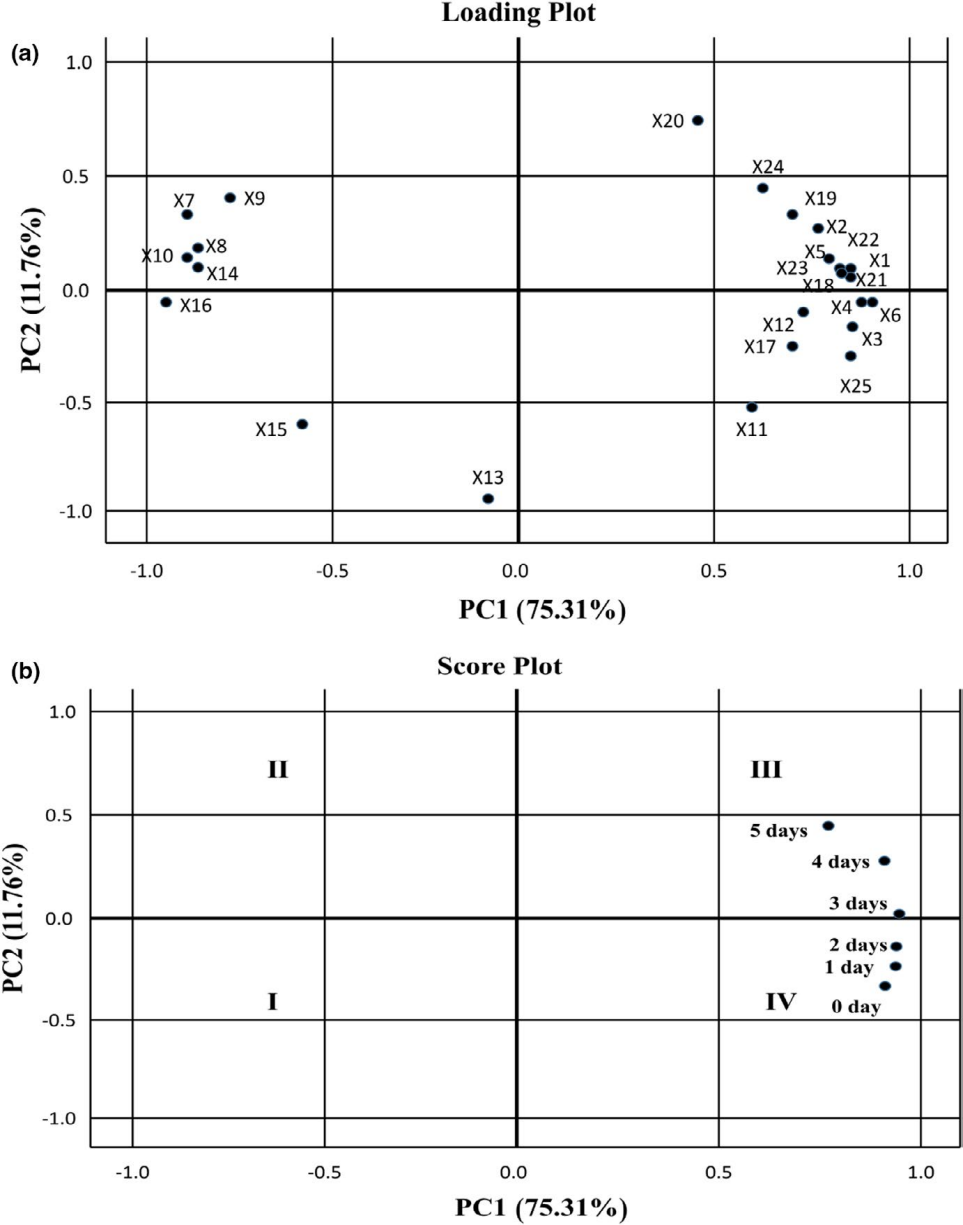
Principle component analysis loading plot (a) and score plot (b) describing the relationship among different variables of flaxseed during germination. Abbreviations: X1: DPPH; X2: FRAP; X3: ABTS; X4: IOT; X5: total phenolics; X6: flavonoids; X7: total CLs; X8: SDG; X9: p‐CouAG; X10: FeAG; X11: campesterol; X12: stigmasterol; X13: β‐sitosterol; X14: Δ5‐avenasterol; X15: cycloartenol; X16: γ‐tocopherol; X17: α‐tocopherol; X18: protocatechuic acid; X19: 4‐hydroxybenzoic acid; X20: syringic acid; X21: p‐coumaric acid; X22: ferulic acid; X23: caffeic acid; X24: vanillin; X25: gallic acid

All data obtained from the germinated flaxseed were subjected to principal component analysis (PCA). The first two principal components explained 87.07% of the total variation (PC1 = 75.31% and PC2 = 11.76%). As shown in Figure 7(a), PC1 was highly positive affected by DPPH, FRAP, ABTS, OTI, total phenolic acids, flavonoids, α‐tocopherol, stigmasterol, protocatechuic acid, *p*‐coumaric acid, ferulic acid, caffeic, and gallic acids, but negatively impacted by the total CLs, SDG, FeAG, *p*‐CouAG, Δ5‐avenasterol, and γ‐tocopherol. PC2 was primarily positively correlated with the vanillin, syringic acid, and 4‐hydroxybenzoic acid, but negatively impacted by *β*‐sitosterol, campesterol, and cycloartenol. As depicted in Figure 7(b), it showed the score plot between PC1 and PC2. Flaxseed placed in quadrant III (4–5 days of germination samples) had higher DPPH, ABTS, FRAP values, total phenolics, 4‐hydroxybenzoic acid, syringic acid, ferulic acid, *p*‐coumaric acid, caffeic acid, and vanillin in comparison with those placed in quadrant IV (0–2 days of germination). However, the flaxseed placed in Quadrant III and IV had low total CLs, SDG, *p*‐CouAG, FeAG, *β*‐sitosterol, Δ5‐avenasterol, cycloartenol, and γ‐tocopherol contents. The flaxseed obtained from 5 days of germination (placed in Quadrant III) showed the most distinctive behavior among all samples with the highest antioxidant activities (DPPH, ABTS, and FRAP), which could be attributed to the abundant ferulic acid, *p*‐coumaric acid, caffeic acid, and gallic acid.

## 4 CONCLUSION

In conclusion, the ACEW promotes the growth of flaxseed sprouts and simultaneously suppresses the preferential degradation of ALA in triglycerides of flaxseed during germination when compared with that of TW. Moreover, the accumulation of bioactive lipid concomitants in flaxseed sprouts, including lignans, phenolic acids, flavonoids, tocopherols, and phytosterols, are further favorably induced by ACEW following appropriate germination in comparison with those by TW, which is parallel with the optimized thermal oxidation stability and antioxidant capacities of flaxseed and flaxseed oil. Therefore, the application of germination for flaxseed especially induced by ACEW can be favorable for further improving the nutritional and functional properties of flaxseed.

## CONFLICTS OF INTEREST

The authors declare no competing financial interest.

## AUTHOR CONTRIBUTIONS

**Shasha Huang:** Conceptualization (equal); Data curation (supporting); Formal analysis (equal); Methodology (equal); Writing‐original draft (lead). **Haicheng Zhang:** Data curation (lead); Formal analysis (equal); Validation (lead). **Xiaopeng Qin:** Investigation (equal); Methodology (equal); Software (lead). **Chengzhen Nie:** Methodology (lead); Software (supporting). **Xiao Yu:** Writing‐review & editing (lead). **Deng Qianchun:** Supervision (lead).

## Supporting information

Fig. S1
Table S1
Table S2

## References

[fsn32538-bib-0001] Barthet, V. J. , Klensporf‐Pawlik, D. , & Przybylski, R. (2014). Antioxidant activity of flaxseed meal components. Canadian Journal of Plant Science, 94(3), 593–602. 10.4141/cjps2013-018

[fsn32538-bib-0002] Brühl, L. , Matthäus, B. , Fehling, E. , Wiege, B. , Lehmann, B. , Luftmann, H. , Bergander, K. , Quiroga, K. , Scheipers, A. , Frank, O. , & Hofmann, T. (2007). Identification of bitter off‐taste compounds in the stored cold pressed linseed oil. Journal of Agricultural and Food Chemistry, 55(19), 7864–7868. 10.1021/jf071136k 17715895

[fsn32538-bib-0003] Chen, L. , Tan, J. T. G. , Zhao, X. , Yang, D. Y. , & Yang, H. S. (2019). Energy regulated enzyme and non‐enzyme‐based antioxidant properties of harvested organic mung bean sprouts (Vigna radiata). LWT‐Food Science and Technology, 107, 228–235. 10.1016/j.lwt.2019.03.023

[fsn32538-bib-0004] Choo, W. S. , Birch, J. , & Dufour, J. P. (2007). Physicochemical and quality characteristics of cold‐pressed flaxseed oils. Journal of Food Composition and Analysis, 20(3‐4), 202–211. 10.1016/j.jfca.2006.12.002

[fsn32538-bib-0005] Cong, Y. X. , Zheng, M. M. , Huang, F. H. , Liu, C. S. , & Zheng, C. (2020). Sinapic acid derivatives in microwave‐pretreated rapeseeds and minor components in oils. Journal of Food Composition and Analysis, 87. 10.1016/j.jfca.2019.103394

[fsn32538-bib-0006] Deng, Q. C. , Yu, X. , Ma, F. L. , Xu, J. Q. , Huang, F. H. , Huang, Q. D. , & Sheng, F. (2018). Comparative analysis of the *in‐vitro* antioxidant activity and bioactive compounds of flaxseed in China according to variety and geographical origin. International Journal of Food Properties, 20, S2708–S2722. 10.1080/10942912.2017.1402029

[fsn32538-bib-0007] Gui, B. , Shim, Y. Y. , Datla, R. S. S. , Covello, P. S. , Stone, S. L. , & Reaney, M. J. T. (2012). Identification and quantification of cyclolinopeptides in five flaxseed cultivars. Journal of Agricultural and Food Chemistry, 60(35), 8571–8579. 10.1021/jf301847u 22897677

[fsn32538-bib-0008] Hao, J. X. , Wu, T. J. , Li, H. Y. , Wang, W. , & Liu, H. J. (2016). Dual effects of slightly acidic electrolyzed water (SAEW) treatment on the accumulation of gamma‐aminobutyric acid (GABA) and rutin in germinated buckwheat. Food Chemistry, 201, 87–93. 26868552 10.1016/j.foodchem.2016.01.037

[fsn32538-bib-0009] Herchi, W. , Bahashwan, S. , Sebei, K. , Ben Saleh, H. , Kallel, H. , & Boukhchina, S. (2015). Effects of germination on chemical composition and antioxidant activity of flaxseed (*Linum usitatissimum* L) oil. Grasas Y Aceites, 66(1). 10.3989/gya.0463141

[fsn32538-bib-0010] Herchi, W. , Harrabi, S. , Sebei, K. , Rochut, S. , Boukhchina, S. , Pepe, C. , & Kallel, H. (2009). Phytosterols accumulation in the seeds of *Linum usitatissimum L* . Plant Physiology and Biochemistry, 47(10), 880–885. 10.1016/j.plaphy.2009.07.001 19616960

[fsn32538-bib-0011] Jadhav, P. D. , Okinyo‐Owiti, D. P. , Ahiahonu, P. W. K. , & Reaney, M. J. T. (2013). Detection, isolation and characterisation of cyclolinopeptides J and K in ageing flax. Food Chemistry, 138(2–3), 1757–1763. 10.1016/j.foodchem.2012.10.126 23411308

[fsn32538-bib-0012] Lao, Y. W. , Mackenzie, K. , Vincent, W. , & Krokhin, O. V. (2014). Characterization and complete separation of major cyclolinopeptides in flaxseed oil by reversed‐phase chromatography. Journal of Separation Science., 37(14), 1788–1796. 10.1002/jssc.201400193 24788784

[fsn32538-bib-0013] Müller, L. , Theile, K. , & Böhm, V. (2010). In vitro antioxidant activity of tocopherols and tocotrienols and comparison of vitamin E concentration and lipophilic antioxidant capacity in human plasma. Molecular Nutrition and Food Research, 54(5), 731–742. 10.1002/mnfr.200900399 20333724

[fsn32538-bib-0014] Li, K. , Mikola, M. R. , Draths, K. M. , Worden, R. M. , & Frost, J. W. (1999). Fed‐batch fermentor synthesis of 3‐dehydroshikimic acid using recombinant *Escherichia coil* . Biotechnology and Bioengineering, 64(1), 61–73. https://doi.org/10.1002/(SICI)1097‐0290(19990705)64:1<61::AID–BIT7>3.0.CO 10397840

[fsn32538-bib-0015] Li, X. , Li, J. Y. , Dong, S. , Li, Y. F. , Wei, L. P. , Zhao, C. C. , Li, J. Y. , Liu, X. B. , & Wang, Y. T. (2019). Effects of germination on tocopherol, secoisolarlciresinol diglucoside, cyanogenic glycosides and antioxidant activities in flaxseed (*Linum usitatissimum* L.). International Journal of Food Science and Technology, 54(7), 2346–2354. 10.1111/ijfs.14098

[fsn32538-bib-0016] Liu, Q. , Tan, T. S. C. , Yang, H. S. , & Wang, S. F. (2017). Treatment with low‐concentration acidic electrolysed water combined with mild heat to sanitise fresh organic broccoli (Brassica oleracea). LWT‐Food Science and Technology, 79, 594–600. 10.1016/j.lwt.2016.11.012

[fsn32538-bib-0017] Liu, R. , He, X. L. , Shi, J. Q. , Nirasawa, S. , Tatsumi, E. , Li, L. T. , & Liu, H. J. (2013). The effect of electrolyzed water on decontamination, germination and gamma‐aminobutyric acid accumulation of brown rice. Food Control, 33(1), 1–5. 10.1016/j.foodcont.2013.02.008

[fsn32538-bib-0018] Moreau, R. A. , Nyström, L. , Whitaker, B. D. , Winkler‐Moser, J. K. , Baer, D. J. , Gebauer, S. K. , & Hicks, K. B. (2018). Phytosterols and their derivatives: Structural diversity, distribution, metabolism, analysis, and health‐promoting uses. Progress in Lipid Research, 70, 35–61. 10.1016/j.plipres.2018.04.001 29627611

[fsn32538-bib-0019] Muñoz, P. , & Munné‐Bosch, S. (2019). Vitamin E in plants: biosynthesis, transport, and function. Trends in Plant Science, 24(11), 1040–1051. 10.1016/j.tplants.2019.08.006 31606282

[fsn32538-bib-0020] Nieva‐Echevarría, B. , Goicoechea, E. , & Guillén, M. D. (2017). Behaviour of non‐oxidized and oxidized flaxseed oils, as models of omega‐3 rich lipids, during *in vitro* digestion. Occurrence of epoxidation reactions. Food Research International Journal, 97, 104–115. 10.1016/j.foodres.2017.03.047 28578030

[fsn32538-bib-0021] Nikiforidis, C. V. (2019). Structure and functions of oleosomes (oil bodies). Advances in Cilloid and Interface Science, 274, 102039. 10.1016/j.cis.2019.102039 31683192

[fsn32538-bib-0022] Parikh, M. , Maddaford, T. G. , Austria, J. A. , Aliani, M. , Netticadan, T. , & Pierce, G. N. (2019). Dietary flaxseed as a strategy for improving human health. Nutrients, 11(5), 1171. 10.3390/nu11051171 PMC6567199 31130604

[fsn32538-bib-0023] Pilar, B. , Güllich, A. , Oliveira, P. , Ströher, D. , Piccoli, J. , & Manfredini, V. (2017). Protective role of flaxseed oil and flaxseed lignan secoisolariciresinol diglucoside against oxidative stress in rats with metabolic syndrome. Journal of Food Science, 82(12), 3029–3036. 10.1111/1750-3841.13964 29083494

[fsn32538-bib-0024] Ramsay, A. , Fliniaux, O. , Quéro, A. , Molinié, R. , Demailly, H. , Hano, C. , Paetz, C. , Roscher, A. , Grand, E. , Kovensky, J. , Schneider, B. , & Mesnard, F. (2017). Kinetics of the incorporation of the main phenolic compounds into the lignan macromolecule during flaxseed development. Food Chemistry, 217, 1–8. 10.1016/j.foodchem.2016.08.039 27664601

[fsn32538-bib-0025] Shi, H. L. , Nam, P. K. , & Ma, Y. F. (2010). Comprehensive profiling of isoflavones, phytosterols, tocopherols, minerals, crude protein, lipid, and sugar during soybean (*Glycine max*) germination. Journal of Agricultural and Food Chemistry, 58(8), 4970–4976. 20349962 10.1021/jf100335j

[fsn32538-bib-0026] Shim, Y. Y. , Gui, B. , Arnison, P. G. , Wang, Y. , & Reaney, M. J. T. (2014). Flaxseed (*Linum usitatissimum L*.) bioactive compounds and peptide nomenclature: A review. Trends in Food Science and Technology, 38(1), 5–20. 10.1016/j.tifs.2014.03.011

[fsn32538-bib-0027] Shintani, D. , & DellaPenna, D. (1998). Elevating the vitamin E content of plants through metabolic engineering. Science, 282(5396), 2098–2100. 10.1126/science.282.5396.2098 9851934

[fsn32538-bib-0028] Singh, A. (2013). Sitosterol as an antioxidant in frying oils. Food Chemistry, 137(1–4), 62–67. 10.1016/j.foodchem.2012.10.008 23199991

[fsn32538-bib-0029] Suri, K. C. , Singh, B. , Kaur, A. , Yadav, M. P. , & Singh, N. (2020). Influence of microwave roasting on chemical composition, oxidative stability and fatty acid composition of flaxseed (*Linum usitatissimum L*.) oil. Food Chemistry, 326, 126974. 32413759 10.1016/j.foodchem.2020.126974

[fsn32538-bib-0030] Touré, A. , & Xueming, X. (2010). Flaxseed lignans: source, biosynthesis, metabolism, antioxidant activity, bio‐active components, and health benefits. Comprehensive Reviews in Food Science and Food Safety, 9(3), 261–269. 10.1111/j.1541-4337.2009.00105.x 33467817

[fsn32538-bib-0031] Vogt, T. (2010). Phenylpropanoid biosynthesis. Molecular Plant, 3(1), 2–20. 10.1093/mp/ssp106 20035037

[fsn32538-bib-0032] Wanasundara, P. K. J. P. D. , Wanasundara, U. N. , & Shahidi, F. (1999). Changes in flax (*Linum usitatissimum* L.) seed lipids during germination. Journal of the American Oil Chemists Society, 76(1), 41–48. 10.1007/s11746-999-0045-z

[fsn32538-bib-0033] Wang, H. , Qiu, C. S. , Abbasi, A. M. , Chen, G. , You, L. J. , Li, T. , Fu, X. , Wang, Y. F. , Guo, X. B. , & Liu, R. H. (2015). Effect of germination on vitamin C, phenolic compounds and antioxidant activity in flaxseed (*Linum usitatissimum* L.). International Journal of Food Science and Technology, 50(12), 2545–2553. 10.1111/ijfs.12922

[fsn32538-bib-0034] Wang, H. , Wang, J. H. , Guo, X. B. , Brennan, C. S. , Li, T. , Fu, X. , Chen, G. , & Liu, R. H. (2016). Effect of germination on lignan biosynthesis, and antioxidant and antiproliferative activities in flaxseed (*Linum usitatissimum* L.). Food Chemistry, 205, 170–177. 10.1016/j.foodchem.2016.03.001 27006228

[fsn32538-bib-0035] Xiang, Q. S. , Liu, X. F. , Liu, S. N. , Ma, Y. F. , Xu, C. Q. , & Bai, Y. H. (2019). Effect of plasma‐activated water on microbial quality and physicochemical characteristics of mung bean sprouts. Innovative Food Science and Emerging Technologies, 52, 49–56. 10.1016/j.ifset.2018.11.012

[fsn32538-bib-0036] Xie, Y. , Wei, F. , Xu, S. L. , Wu, B. F. , Zheng, C. , Lv, X. , Wu, Z. Y. , Chen, H. , & Huang, F. H. (2018). Profiling and quantification of lipids in cold‐pressed rapeseed oils based on direct infusion electrospray ionization tandem mass spectrometry. Food Chemistry, 285, 194–203. 10.1016/j.foodchem.2019.01.146 30797335

[fsn32538-bib-0037] Yu, X. , Deng, Q. C. , Tang, Y. H. , Xiao, L. , Liu, L. G. , Yao, P. , Tang, H. , & Dong, X. Y. (2018). Flaxseed oil attenuates hepatic steatosis and insulin resistance in mice by rescuing the adaption to ER stress. Journal of Agricultural and Food Chemistry, 66(41), 10729–10740. 10.1021/acs.jafc.8b03906 30145885

[fsn32538-bib-0038] Yu, X. , Huang, S. S. , Nie, C. Z. , Deng, Q. C. , Zhai, Y. F. , & Shen, R. L. (2020). Effects of atmospheric pressure plasma jet on the physicochemical, functional, and antioxidant properties of flaxseed protein. Journal of Food Science, 85(7), 2010–2019. 10.1111/1750-3841.15184 32529640

[fsn32538-bib-0039] Zhang, J. F. , & Yang, H. S. (2017). Effects of potential organic compatible sanitisers on organic and conventional fresh‐cut lettuce (*Lactuca sativa Var. Crispa L*). Food Control, 72, 20–26. 10.1016/j.foodcont.2016.07.030

[fsn32538-bib-0040] Zou, X. G. , Hu, J. N. , Zhu, X. M. , Wang, Y. F. , & Deng, Z. Y. (2018). Methionine sulfone‐containing orbitides, good indicators to evaluate oxidation process of flaxseed oil. Food Chemistry, 250, 204–212. 10.1016/j.foodchem.2018.01.030 29412912

