# Supplementary material for: The quality and antioxidant elucidation of germinated flaxseed treated with acidic electrolyzed water
Source: Food Sci Nutr. 2021 Sep 10;9(11):6031–46. doi: 10.1002/fsn3.2538 (PMC8565247; doi:10.1002/fsn3.2538)
Supplement: Supplementary file 1 — Fig. S1 Table S1 Table S2 [file FSN3-9-6031-s001.docx]

**Fig.S1 Change in the germination rate of flaxseed during germination. Means with different letters are significantly different at *p*<0.05 level.**

**Fig.S2 Change in the crude protein and total sugars of flaxseed during germination. Means with different letters are significantly different at *p*<0.05 level.**

**Table S1 Change in the individual CL of flaxseed during germination.**

| CLs  (mg/100 g) | Germination treatment | | | | | | | | | | |
| --- | --- | --- | --- | --- | --- | --- | --- | --- | --- | --- | --- |
|  | Control | TW | | | | | ACEW | | | | |
|  | 0 | 1 | 2 | 3 | 4 | 5 | 1 | 2 | 3 | 4 | 5 |
| CL-A | 13.05±0.59h | 11.80±0.31f | 6.32±0.12d | 5.68±0.23c | 5.48±0.23c | 3.95±0.12a | 12.99±0.89g | 7.52±0.54e | 6.48±0.32d | 5.72±0.32c | 4.73±0.13b |
| CL-N | 4.60±0.13g | 4.30±0.22f | 2.37±0.12d | 1.44±0.09c | 0.70 ±0.03b | 0.40±0.01a | 4.51±0.02fg | 3.07±0.02e | 1.54±0.02c | 0.90±0.03b | 0.42±0.03a |
| CL-O | 19.79±0.65h | 15.02±0.063g | 9.71±0.41f | 7.57±0.25d | 6.85±0.34c | 3.64±0.22a | 15.66±0.86g | 11.06±0.48g | 8.57±0.19e | 7.10±0.27d | 4.33±0.14b |
| CL-M | 6.12±0.28a | 5.94±0.43a | 4.63±0.37b | 4.11±0.22c | 3.15±0.16d | 1.50±0.09a | 6.02±0.14a | 5.08±0.33b | 4.51±0.26c | 3.16±0.17d | 1.69±0.11a |
| **CL: Cyclolinopeptide; Means with different letters are significantly different at *p*<0.05 level.** | | | | | | | | | | | |
